# Ultrasonic Spray Coating to Optimize Performance of Bio-Electrochemical Systems

**DOI:** 10.3390/nano13222926

**Published:** 2023-11-10

**Authors:** Giacomo Spisni, Giulia Massaglia, Fabrizio C. Pirri, Stefano Bianco, Marzia Quaglio

**Affiliations:** 1 Department of Applied Science and Technology, Politecnico di Torino, 10129 Turin, Italy; giacomo.spisni@iit.it (G.S.); candido.pirri@polito.it (F.C.P.); stefano.bianco@polito.it (S.B.); 2 Centre for Sustainable Future Technologies @ PoliTo, Istituto Italiano di Tecnologia, 10146 Turin, Italy

**Keywords:** nanostructured layer, ultrasonic spray coating, intrinsically conductive polymer, anode electrode, bioelectrochemical devices

## Abstract

This work investigates the optimization of carbon-based electrodes employed in bio-electrochemical systems (BES) through the deposition of nanostructured layers of poly(3,4-ethylene-dioxy-thiophene) poly(styrene-sulfonate) (PEDOT:PSS) on commercial carbon paper electrodes via ultrasonic spray coating (USC). This innovative application of USC demonstrated that uniform and controlled depositions of PEDOT:PSS can be successfully performed on carbon-based electrodes. To this end, the morphology and spatial uniformity of depositions were verified via scanning electron microscopy and Raman spectroscopy. Electrochemical characterizations of fabricated electrodes demonstrated a more than two-fold increase in the electrochemical active surface area with respect to bare carbon paper. A lab-scale experiment on BES was performed, selecting microbial fuel cells (MFCs) as the reference devices. Devices featuring USC-deposited PEDOT:PSS electrodes showed a three-fold-higher energy recovery with respect to control cells, reaching a maximum value of (13 ± 2) J·m^−3^. Furthermore, the amount of PEDOT:PSS required to optimize MFCs’ performance is in line with values reported in the literature for other deposition methods. In conclusion, this work demonstrates that USC is a promising technique for application in BES.

## 1. Introduction

Among the different classes of bio-electrochemical systems, microbial fuel cells (MFC) and microbial electrolysis cells (MEC) have received great attention in the ever-growing context of sustainable energy production. The crucial feature of both MFC and MEC systems can be identified in the anode electrode, on which an electroactive biofilm must develop [1,2,3]. The electrical activity of the biofilm plays a key role in defining the performance of the bio-electrochemical devices. Indeed, electroactive biofilms show the ability to directly convert chemical energy, trapped in organic matter known as fuel, into electrical energy, acting as a biocatalyst for the oxidation reaction of fuel [1,2,3,4,5,6,7,8,9,10,11,12,13]. In light of that, several works in the literature have focused on the design and fabrication of anode electrodes, implementing different strategies to improve the properties of electrodes’ surface in terms of hydrophilicity, porosity, and electrical conductivity [4,5,6,7,8,9,10,11,12,13]. Indeed, it is widely acknowledged that there is a direct correlation between the surface area of the anode electrode, biofilm formation and growth, and the consequent optimisation of device performance [10,14,15,16]. With the main purpose of improving the electrical conductivity of the anodes while preserving their inherent continuous porosity, many works in the literature propose the deposition of a poly(3,4-ethylene-dioxy-thiophene) poly(styrene-sulfonate)(PEDOT:PSS) layer on standard carbon substrates [11,13,14,17,18,19]. PEDOT:PSS is a well-known intrinsically conductive polymer [20,21] that has been successfully applied in a wide range of industrial applications, including biomedicine and energy conversion and storage [21,22,23]. In such applications, PEDOT:PSS represented an ideal choice thanks to its stability in water-based solutions, its superior physical–chemical properties, its bio-compatibility and non-cytotoxicity, and its processability through a wide range of deposition techniques [20,21]. All these features make PEDOT:PSS the ideal candidate for optimizing the surface area of anode electrodes in BES. To this end, indeed, the presence of PEDOT:PSS enabled the improvement of anode performance through the coupling of the continuous electrical conductivity of this intrinsically conductive polymer with the porosity of standard carbon-based materials, optimizing their surfaces to host electroactive biofilms. To reach this goal, several techniques for the processing of PEDOT:PSS were proposed, such as electrochemical methods [11,14,24,25] and dip coating [19]. Such techniques may pose limitations when scaled to large-area electrodes, requiring the use of high-current appliances and large volumes of solution precursors. To overcome all these limitations, in the present work, an ultrasonic spray-coating (USC) technique was proposed for the deposition of a nanostructured layer of PEDOT:PSS, able to achieve a uniform coverage of the three-dimensional surfaces of carbon electrodes (i.e., carbon paper, CP). The USC technique is based on the dispersion of the spraying ink via high-frequency vibrations induced at the spraying nozzle [26,27]. It is a versatile technology for the fabrication of thin nano-coatings and it can be easily scaled up for deposition on large areas with optimal uniformity and conformity. In addition, USC enables precise control of the spatial uniformity of the deposited layer and selection of the amount of material to be deposited [26]. USC has been widely used in fuel cells for the deposition of catalyst materials [28,29,30] and in organic electronics applications [31], highlighting the potential of USC for the fabrication of smooth and uniform thin films on plain substrates [27,31,32]. 

In the present work, new anode electrodes for MFCs were fabricated by depositing nano-coatings of PEDOT:PSS using the USC technique. Thanks to the key features offered by USC, uniform coatings were optimized on the surfaces of carbon-based materials used as the conductive backbone of the electrodes in MFCs. In this scenario, the application of USC as a methodology to improve the performance of anodes in MFCs has been anticipated in our previous work [33]. In this work, employing USC, the detailed optimization of nanostructured layers of PEDOT:PSS on the anode electrodes is reported. The role of the deposition parameters on the uniformity of the final coating is investigated in detail. Among the parameters, a careful analysis is conducted to best understand the role of the amount of PEDOT:PSS deposited. Three different values of the amount of PEDOT:PSS were selected and analysed. The electrochemical active surface area was calculated to appreciate the performance of the fabricated electrodes. The best–performing anodes showed a (2.6 ± 0.2)-times increase over the reference bare carbon paper.

In addition, Raman spectroscopy was performed to analyse and map the spatial distribution of such layers. This was possible by correlating the presence of PEDOT:PSS with the intensity of a characteristic Raman peak (1437 cm^−1^, Cα = Cβ symmetric stretching vibrations inside PEDOT:PSS).

Finally, since the properties of the newly developed anodes directly impact the overall performance of single-chamber microbial fuel cells (SCMFCs), the electrodes have been tested in devices to assess their performance and stability. In terms of energy recovery, SCMFCs featuring the PEDOT:PSS nanostructured layer achieved, at best, (13 ± 2) J·m^−3^, which is approximately three-times higher compared to the control cells at (4.3 ± 0.5) J·m^−3^.

## 2. Materials and Methods

### 2.1. Anode Fabrication via Ultrasonic Spray Coating (USC)

All anode electrodes created by depositing a PEDOT:PSS nanostructured layer on bare carbon paper were fabricated using ultrasonic spray coating (Nadetech Innovations, Noáin, Spain). This technique offers the great advantage of directly depositing the nanostructured layer on the carbon paper with good uniformity and adhesion, without the need to use binders.

Anode electrodes were fabricated from commercial carbon paper (AvCarb, Lowell, MA, USA). The material, used without pre-treatments, was cut into 30 × 30 mm^2^ square pieces. Spraying solutions (i.e., inks) for the deposition of the nanostructured coatings were prepared by mixing poly(3,4-ethylene-dioxy-thiophene) poly(styrene-sulfonate) (PEDOT:PSS, 1.3 wt.% water dispersion, purchased from Sigma Aldrich, Darmstadt, Germany Germany), and Milli-Q de-ionized water (Merck Millipore, Darmstadt, Germany). Each substrate was held in place on the heated deposition plate by a silicone mask and vacuum suction. To maximize the spatial uniformity of the nanostructured layer, the spray nozzle was kept in motion following a specific pattern. The pattern was repeated multiple times so as to achieve the target amount of deposited material. The overall duration of the process ranged from 2 to 6 min.

As discussed in a previous work [33], the ink was prepared by diluting the as-purchased PEDOT:PSS aqueous dispersion (0.5 wt.% PEDOT, 0.8 wt.% PSS) in de-ionized water (2:8 volume ratio), resulting in a final solution with a PEDOT concentration equal to 1 mg/mL and a PSS concentration equal to 1.6 mg/mL. The prepared solution was stored at 4 °C and it was processed with magnetic stirring for 1 h prior to use. The high dilution ensured the precise modulation of the final amount of PEDOT deposited and a high spatial uniformity.

To obtain a uniform PEDOT:PSS nanostructured layer, the following process parameters were optimized: the flow rate, the piezoelectric nozzle operation power and frequency, the nozzle-to-plate distance, and the deposition plate temperature.

In previous works in the literature, it has been shown that the optimal amount of PEDOT:PSS to achieve high-performance MFC anodes is between 20 μg/cm^2^ and 100–300 μg/cm^2^ [11,19]. In addition, it was highlighted that an excessive quantity of deposited material might increase the internal resistance of the anode, thus being detrimental for MFC performance [11,17].

**Figure 1:**
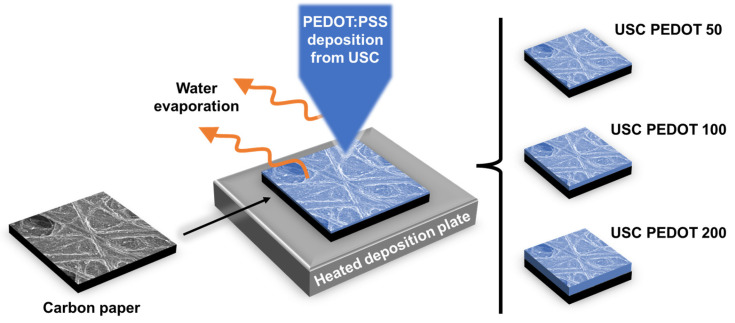
Schematic representation of the anodes fabrication process by USC. Different electrodes were fabricated by varying the amount of the deposited material.

With the main purpose of investigating how USC-deposited PEDOT:PSS can affect the performance of anode electrodes, in this work electrodes were fabricated by depositing nanostructured layers with different amounts of PEDOT:PSS. As represented in Figure 1, three different sets of anode electrodes were fabricated and compared: (i) USC PEDOT 50, containing 50 μg/cm^2^ of PEDOT; (ii) USC PEDOT 100 made of 100 μg/cm^2^ of PEDOT; and (iii) USC PEDOT 200 based on 200 μg/cm^2^ of PEDOT. All fabricated anode electrodes were directly compared with a control reference electrode obtained from bare carbon paper. For each set of anodes, multiple identical electrodes were fabricated to test the reproducibility of the process and to obtain a number of replicates sufficient to perform all the planned characterizations.

### 2.2. Characterization Techniques

#### 2.2.1. Morphological and Physical–Chemical Characterizations of Electrodes

A field emission scanning electron microscope (FESEM, ZEISS Supra 40, Carl Zeiss AG, Oberkochen, Germany) equipped with an energy-dispersive X-ray (EDX) detector was used to characterize the surface morphology of anode electrode.

Raman spectroscopy (Renishaw InVia Reflex spectrometer, λ_ex_ = 532 nm) was used to investigate the surface of developed anodes. Attention has been paid to the presence of PEDOT:PSS through the presence of its typical fingerprint peaks. In addition, through the acquisition of Raman spectra at different areas of the electrode surface, it was possible to assess the spatial uniformity of the deposition. To this end, Raman maps were acquired to characterize 300 × 250 μm^2^ areas of the electrode surface. Specifically, a map consisted of a matrix of 31 × 17 spatially resolved Raman spectra, each composing one pixel of the map. With the aid of analysis software, a false-colour Raman map was obtained by calculating for each pixel the ratio of the selected characteristic peaks of PEDOT:PSS to those of carbon.

#### 2.2.2. Cyclic Voltammetry Characterizations of Electrodes

Cyclic voltammetry (CV) characterizations were performed to assess the performance of fabricated electrodes through a PalmSens 4 (PalmSens BV, Houten, The Netherlands) potentiostat. CV characterizations were performed with a three-electrode electrochemical cell. Fabricated anodes were used as a working electrode, silver/silver chloride (Ag/AgCl) served as reference electrode, and a Pt wire acted as a counter electrode. A water-based solution containing potassium hexacyanoferrate (1 mM) and sodium sulphate (100 mM) was used as an electrolyte. CV voltammograms were obtained by scanning the potential from −0.5 V to 0.9 V with a scan rate of 100 mV/s. 

CV characterizations were also performed to quantify the electrochemical active surface area (EASA) of the PEDOT:PSS nanostructured layer. Then, EASA was indirectly determined by analysing the CV curves considering the following Equation (1), known as Matsuda’s equation:

(1) $$i_{P}=0.4463\times 10^{-3}\cdot \sqrt{\frac{n^{3}{\times F}^{3}{\times c}_{r}^{2}\times D}{R\times T}}\times A\times \sqrt{\theta }$$

where n = 1 is the number of electrons transferred, F ≈ 96,485 C·mol^−1^ is Faraday’s constant, c_r_ = 10^−3^ mol·L^−1^ is the initial potassium hexacyanoferrate concentration, D = 5.79 × 10^−6^ cm^2^·s^−1^ is the diffusion coefficient of potassium hexacyanoferrate, R ≈ 8.314 J·mol^−1^·K^−1^ is the gas constant, T = 293 K is the electrolyte solution temperature. Additionally, i_p_ (A) is the peak current measured from CV curves during the oxidation of potassium hexacyanoferrate and θ (mV·s^−1^) is the scan rate employed for the CV acquisition, namely 10, 20, and 50 mV·s^−1^. Finally, A (cm^2^) is the electrochemical active surface area to be determined.

#### 2.2.3. SCMFCs Fabrication and Electrical and Electrochemical Characterizations

**Figure 2:**
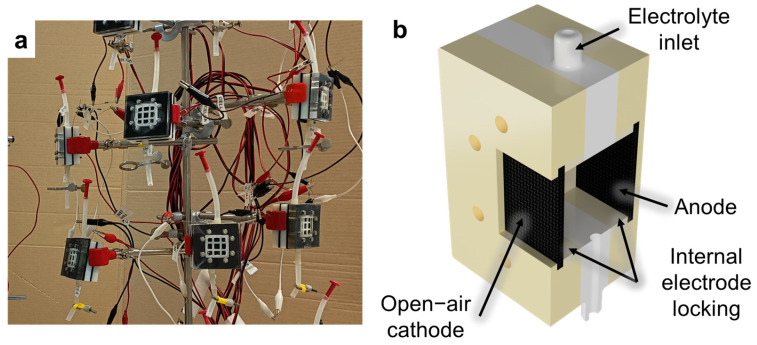
(**a**) Picture and (**b**) simplified cross-section view of some of the MFC devices employed to assess performances of fabricated electrodes.

As described in our previous works [15,16,32,34], open-air cathode single-chamber microbial fuel cells (SCMFCs) were used (Figure 2a). These devices are characterized by a single electrochemical chamber without a membrane and have the electrodes immersed in the same electrolyte (as sketched in Figure 2b). A fixed distance between anode and cathode electrodes was ensured by an intermediate compartment with inlet and outlet holes and an upper opening for inserting the reference electrode. In this configuration, the reference electrode was immersed in the cell at a fixed distance between the anode and cathode. The total internal volume of the cell was 12.5 cm^3^ and the geometric area of both anode and cathode electrodes was equal to 5.76 cm^2^. A water-based electrolyte, containing sodium acetate (C_2_H_3_NaO_2_, 1 g/L) as a carbon energy source, ammonium chloride (NH_4_Cl, 0.31 g/L) and potassium chloride (KCl, 0.13 g/L) as nitrogen and minerals sources, and sodium di-hydrogen phosphate (NaH_2_PO_4_, 2.450 g/L) for pH stability, was used. The electrolyte solution, obtained by dissolving the reagents in de-ionized water, was autoclaved prior to use. All these reagents were purchased from Sigma Aldrich (Darmstadt, Germany).

All fabricated electrodes, i.e., USC PEDOT 50, USC PEDOT 100, and USC PEDOT 200, respectively, were tested as anodes in SCMFCs and comparted with electrodes made of bare carbon paper used as reference. Commercial electrodes with a gas diffusion system (from AvCarb, Lowell, MA, USA) were used as the cathode electrodes. They featured a poly-tetra-fluoro-ethylene (PTFE) treatment on the air-facing side, and a carbon-based micro-porous surface coating at the electrolyte side. 

To promote a direct oxygen reduction reaction (ORR) [15,35], a catalyst paste was deposited on the micro-porous surface [15,16,32,33,34,35]. The paste was based on 0.5 mg/cm^2^ of platinum (10 wt% on carbon, from Sigma Aldrich, Darmstadt, Germany) and 3 mg/cm^2^ of Nafion (5 wt%, from Sigma Aldrich, Darmstadt, Germany) acting as a binder. The cathode and anode electrodes were internally held in place and electrically contacted by 3D-printed frames threaded with titanium wire (Goodfellow Cambridge Ltd., Huntingdon, UK).

A multichannel data acquisition unit (Keysight 34970A, Agilent Technologies, Santa Clara, CA, USA) was used to monitor the output voltage for all SCMFCs, thus evaluating the overall performance of the devices. The experiment for each deposition condition was conducted in triplicate.

A mixed consortium was obtained from marine sediment collected in La Spezia (Italy). It was used to inoculate the MFCs. In agreement with what was discussed in previous works [15,16], during the inoculation phase of the MFCs, an external load of 470 Ω was applied to each cell in order to promote the biofilm formation at the anode surface. Later, during the operative period of the MFCs, the external load to its final value of 1 kΩ was raised.

Energy recovery [3,15,36] was introduced to accurately correlate the MFC performance with the presence of a nanostructured PEDOT:PSS layer onto the anode surface. As reported in different works in the literature [3,15,36], starting from the measured output potential, the average energy recovery parameter was defined by Equation (2):

(2) $$E_{rec}=\frac{\int_{t_{1}}^{t_{2}}P(t)dt}{V_{int}}$$

where E_rec_ (J·m^−3^) is the energy recovery, $\int_{t_{1}}^{t_{2}}P(t)dt$ (J) is the integral of the recovered energy between the initial (t_1_) and final (t_2_) moments associated with each refill, and V_int_ (m^3^) is the cell’s internal volume [3,15,36]. 

Electrochemical impedance spectroscopy (EIS) characterizations were performed during the experiments to investigate the electrochemical interfaces arising inside the cells at the anode electrode, and linear sweep voltammetry (LSV) to assess the overall performance of SCMFCs. Since the cathode electrodes were formally identical for each bio-electrochemical device, EIS characterizations were performed in a three-electrode setup with Ag/AgCl as a reference electrode, thus leading to better investigation of the anode interfaces arising in MFCs. EIS characterization was obtained by imposing an AC sinusoidal signal with a 10 mV amplitude and frequency ranging from 150 kHz to 200 mHz. For LSV, a two-electrode configuration was applied to sweep the anodic potential from open-circuit voltage (OCV) to short-circuit voltage (0 V) at a voltage scan rate of 0.1 V·s^−1^.

## 3. Results and Discussion

### 3.1. Morphological and Physical–Chemical Characterizations

**Figure 3:**
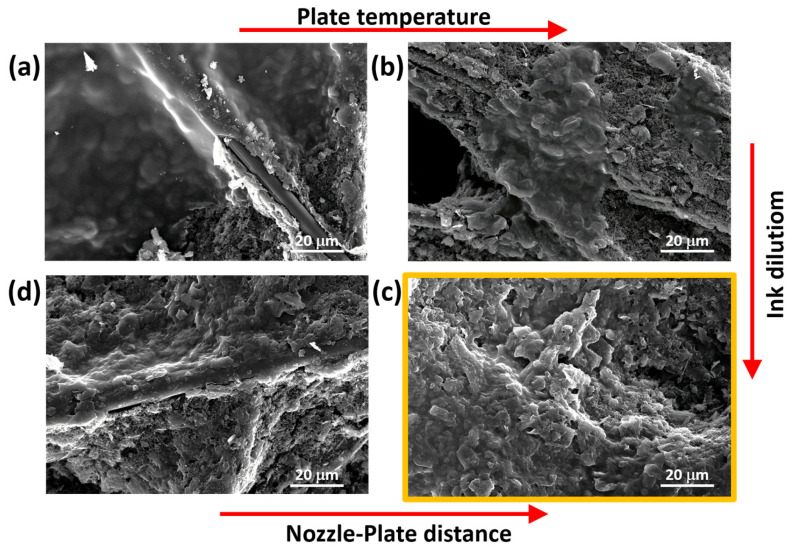
FESEM images of samples prepared to optimize the deposition parameters: comparison of (**a**,**b**) provides information on the role of the temperature; comparison of (**c**,**d**) allows us to appreciate the impact of the nozzle-plate distance; comparison of (**b**,**c**) demonstrates the role of ink dilution.

Given the good level of automation offered by the USC equipment used in this work, the frequency of the piezo oscillator was automatically set to 88–89 kHz. Fixing that frequency, a power setpoint of 2 W resulted in the most appropriate conditions to ensure the ejection of evenly dispersed ink droplets from the nozzle and the optimal stability of the ejected flow. Indeed, a lower value of the flow rate rendered the ejected flow unstable, while increasing it caused the surface of the samples to become wet, making it difficult for the solvent to evaporate properly. The USC deposition of the as-purchased solution using the aforementioned parameters is shown in Figure 3a. For the preparation of that sample, the deposition plate was set at the standard value of 40 °C, and the nozzle-plate distance was fixed at 10 cm.

The morphology of the samples fabricated with the starting set of parameters is characterized by agglomerates as highlighted in Figure 3a. In order to improve the uniformity of the PEDOT:PSS nano-coating, the temperature of the deposition plate was then tested. It was increased from 40 °C to 80 °C, and the morphology changed as shown in Figure 3b. For both of the temperatures, the proposed FESEM pictures show the tendency of PEDOT:PSS to accumulate, leaving part of the carbon paper substrate uncovered. Nonetheless, it is possible to observe a correlation between the dimensions of material clusters and temperature. A lower temperature (40 °C) gave rise to wider agglomerates (hundreds of micrometres), while a high temperature (80 °C) produced smaller, more dispersed agglomerates thanks to a more effective removal of solvent during the process. Limited solvent evaporation favours ink drops’ coalescence driven by surface tension effects, thus causing an inhomogeneous distribution of the deposited materials on the sample’s surface that results in the formation of wider agglomerates. In conclusion, to obtain a more uniform and nanostructured layer, a hotplate kept at 80 °C resulted in the optimal condition. Higher temperatures appeared unnecessary, also keeping in mind material degradation.

Further improvement of the uniformity of the PEDOT:PSS nano-coating can be obtained working on the dilution of the solution. Starting from the as-purchased solution, diluted inks were developed, adding water as the solvent. Indeed, a less concentrated solution is expected to reduce the effective amount of deposited material per unit area. The final amount of PEDOT:PSS to be deposited can be selected by increasing the deposition time. Figure 3b can thus be compared with Figure 3c to appreciate the effect of the dilution, keeping all the other parameters unchanged. The resulting dilution was effective in providing more uniformly distributed depositions as shown in Figure 3c. Finally, the nozzle-plate distance has been investigated. Figure 3c refers to a distance of 10 cm, while Figure 3d shows the effect of a distance between the nozzle and the deposition plate reduced to 8 cm. The comparison of the two pictures clearly shows that keeping the distance at 10 cm allows the optimal deposition condition. Indeed, a reduced nozzle-plate distance determined the formation of large material clusters and large uncovered areas. 

**Figure 4:**
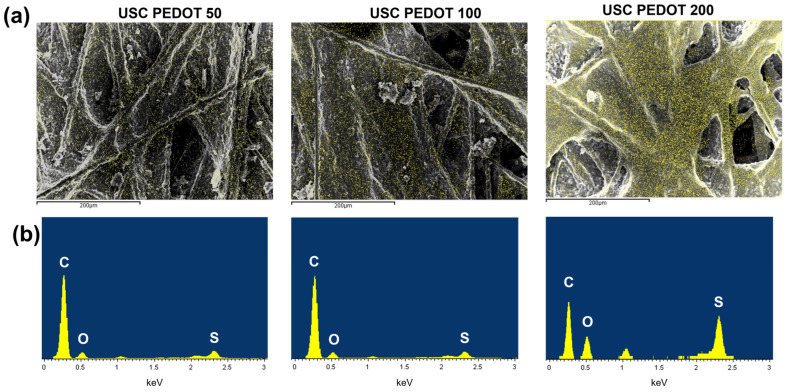
(**a**) FESEM images of the fabricated anodes’ surface (grey scale), overlapped with a map of the spatial distribution of sulphur (yellow pixels) obtained via EDX (sulphur Kα peak at 2.307 keV). (**b**) Corresponding EDX spectra, where the intensities of Kα peaks of carbon, oxygen, and sulphur provide information on their relative abundance. The USC PEDOT 200 image was modified and reprinted from [33] under a CC BY 4.0 license.

The optimal amount of PEDOT:PSS to be deposited by USC on carbon-based electrodes for application as anodes in SCMFCs was then investigated in this work. At first, to gain knowledge on this parameter, the morphology and elemental composition of fabricated electrodes surface has been analysed via FESEM and EDX, respectively. Figure 4a compares FESEM images of the surface of samples obtained from different deposition times as stated in Section 2. EDX maps also overlap to these images, as indicated by the yellow pixels that refer to sites where sulphur was detected (Kα sulphur characteristic emission). Indeed, traces of elemental sulphur on fabricated electrodes can be associated with PEDOT:PSS.

Observing Figure 4a, as the deposited material amount increased, the PEDOT:PSS nanostructured layer became more uniformly distributed over the surface. In addition, analysing the USC PEDOT 200 electrode, it is interesting to notice that the deposited material preferentially accumulated around most superficial fibres before covering the rest of the available surface. Moreover, Figure 4b provides a quantitative comparison of elemental composition via the EDX spectra acquired. When comparing intensities of peaks associated with carbon (Kα at 0.277 keV) and sulphur (Kα at 2.307 keV), it can be observed that the USC PEDOT 200 sample confirmed a sharp increase in sulphur content.

**Figure 5:**
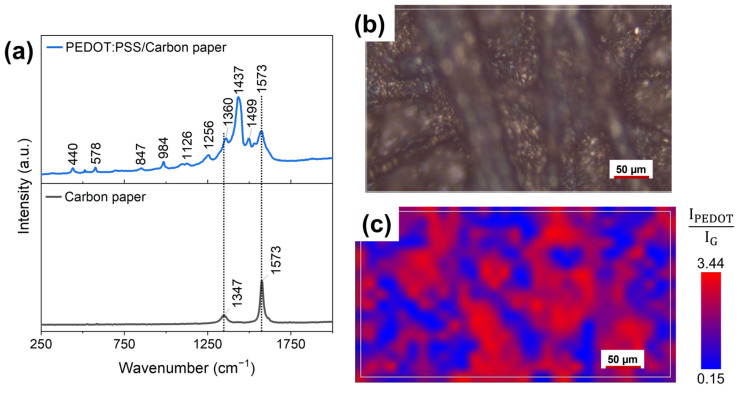
(**a**) Raman spectra acquired on bare carbon paper (black curve) and on the surface of a USC PEDOT 200 fabricated electrode (blue curve). (**b**) Optical microscopy image and (**c**) corresponding false-colour Raman map. The map represents the ratio between the 1437 cm^−1^ and 1573 cm^−1^ peaks of PEDOT:PSS and of carbon, respectively.

With the aim of confirming and complementing the results obtained by EDX measurements, Raman analyses of fabricated electrodes surface were performed. Figure 5a (black curve) presents the reference Raman spectrum of bare carbon paper, featuring the two peaks characteristic of carbon-based materials: the D band (around 1347 cm^−1^), related to the presence of defects, vacancies, and bent sp^2^ bonds in the graphitic structure, and the G band (around 1573 cm^−1^), associated with in-plane vibration of sp^2^ hybridized C-C bonds. Analysing the blue curve in Figure 5a, it was possible to appreciate Raman spectra acquired on the USC PEDOT 200 anode, where a series of additional peaks overlap with those associated with carbon paper. The highest intensity peaks, located at 1256, 1360, and 1437 cm^−1^, can be linked to PEDOT:PSS present on top of the carbon paper substrate. Such peaks can be associated, respectively, with Cα–Cα′ inter-ring stretching, Cβ–Cβ′ stretching, and Cα = Cβ symmetric stretching vibrations [14,37,38]. Other weaker intensity peaks are also compatible with the presence of PEDOT:PSS. These findings are in agreement with those of Kong et al. [37].

The considerations on the uniformity of PEDOT:PSS nanostructured layer were also supported by the analysis of the electrodes’ surface (Figure 5b) via Raman maps (Figure 5c). The false-colour map in Figure 5c shows the relative abundance of PEDOT:PSS composing the nanostructured layer, thus proving its spatial uniformity. The colour grading of each pixel composing the map corresponds to the ratio between the intensity of the PEDOT:PSS characteristic peak at 1437 cm^−1^ (Cα=Cβ symmetric stretching vibrations in PEDOT:PSS) with respect to that of the carbon characteristic peak at 1573 cm^−1^ (G peak). As a result, red areas correspond to regions where the deposited PEDOT:PSS provides a stronger signal than that of the underlying carbon paper. In contrast, the blue areas correspond to carbon-rich regions.

Analysing the Raman map, 527 Raman spectra can be extracted, enabling us to calculate the average intensity of the G-band and D-Band, equal to (421 ± 5) a.u. and (389 ± 5) a.u., respectively. Finally, the R parameter was indirectly determined as the ratio between the intensity of the G-band (I_G_) and the intensity of the D-Band (I_D_), obtaining that R = I_G_/I_D_ = 1.08 ± 0.03. The value of R confirmed the presence of the carbon backbone, covered by a nanostructured layer of PEDOT:PSS.

EDX analysis and Raman spectroscopy offer the evidence of the effectiveness of USC to fabricate nano-coatings able to precisely cover complex surfaces with tri-dimensional features at the micro and nanoscale. Indeed, the results of Figure 5 confirm that the changes in electrodes’ surface morphology are only related to the PEDOT:PSS coating, and they clearly demonstrate that the key features of carbon-paper electrodes are well reproduced.

**Figure 6:**
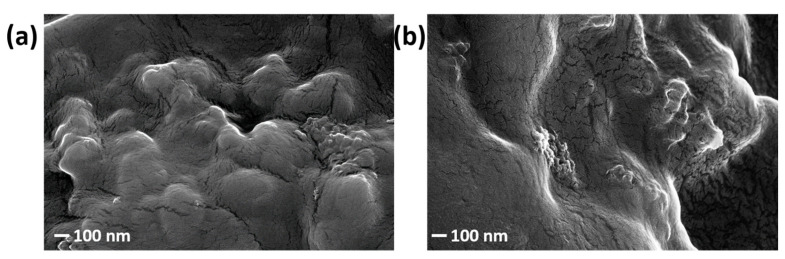
FESEM images of (**a**) USC PEDOT 100 and (**b**) USC PEDOT 200.

After optimization, a final analysis has been dedicated to the nano-scale appearance of the deposited layer. The FESEM analysis of the samples USC PEDOT 100 and USC PEDOT 200 is reported in Figure 6a and Figure 6b, respectively.

The surface of the deposited layer reproduces the underlying carbon paper features, while also showing a diffuse nanoscale roughness. It is interesting to notice that even for the highest amount of PEDOT:PSS deposited, i.e., 200 μg/cm^2^, the surface structure is well reproduced, as well shown in Figure 6b. Once the USC deposition was optimized and the morphology of the nano-structured coatings verified, it was possible to move further in analysing their electrochemical behaviour. 

### 3.2. Electrochemical Characterization on Electrodes

**Figure 7:**
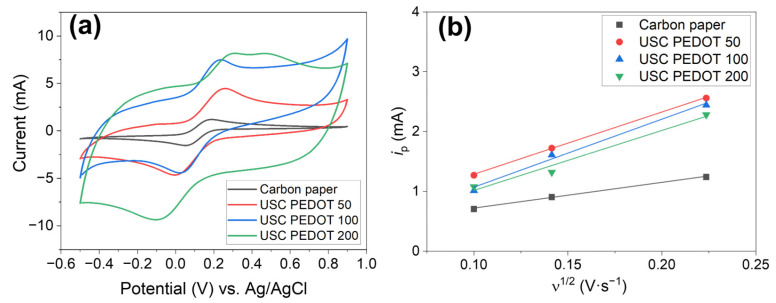
(**a**) Cyclic voltammograms obtained on all investigated electrodes: USC PEDOT 50 (red line), USD PEDOT 100 (blue line), USC PEDOT 200 (green line), and bare carbon paper (black line); (**b**) Determination of electrochemical active surface area, obtained by inverting Matsuda’s equation.

Figure 7a reports cyclic voltammograms obtained for all fabricated electrodes in a hexacyanoferrate electrolyte solution. Comparing these curves, it is possible to perform a rough estimation of the electric double layer capacitance (EDLC) inside the electrochemical cell. Indeed, the potential sweep during CV measurements induces an accumulation of charges at the electrode–electrolyte interface, giving rise to such EDLC. The currents observed during forward and backward scans, which are related to the charge/discharge processes of the EDLC, for a given scan rate can be correlated to the capacity of the EDLC itself. 

It was revealed that the higher the amount of PEDOT:PSS, the greater the maximum current achieved, and so the greater the EDLC value. As a matter of fact, EDLC values could be influenced by the presence of hydrophilic PSS, whose amount also increased linearly with that of PEDOT into the nanostructured layer. It is recognised how PSS is responsible for the increase in surfaces’ wettability and the electrochemical active surface area [14,38]. 

Moreover, it was possible to highlight how USC PEDOT anodes featured a sharper hexacyanoferrate reduction peak with respect to bare carbon paper, indicating an improved electrocatalytic property of USC PEDOT 50, USC PEDOT 100, and USC PEDOT 200 electrodes. 

To better estimate the electrochemical activity of the nanostructured layer obtained by USC, the electrochemical active surface area (EASA) was defined starting from the analysis of CVs. Equation (1) was applied to all the anode electrodes, considering the values of the current i_p_ observed at each scan rate (Figure 7b). The values of i_p_ current, plotted as a function of the square root of the scan rate, provided the EASA values listed in Table 1. With the main aim to demonstrate the improving performances of all investigated samples with respect to the carbon paper reference, a parameter α has been introduced. This parameter is defined as the increase factor of EASA compared with the carbon paper one, as described by the following Equation (3):

(3) $${EASA}_{USC\;PEDOT}=\alpha \cdot {EASA}_{Control}$$

As summarized in Table 1, the results confirmed that all USC PEDOT anode electrodes provided an increase in EASA with respect to the bare carbon paper. The EASA of USC PEDOT 50, close to (15.9 ± 0.3) cm^2^, is 2.4-times higher than the EASA value for carbon paper, equal to (6.6 ± 0.3) cm^2^. USC PEDOT 100 ensured a slight improvement of EASA, achieving a value equal to (17 ± 2) cm^2^, while, on the contrary, USC PEDOT 200 was characterised by a slightly lower electrochemically active area. This result can be explained considering the significantly higher EDLC of USC PEDOT 200 samples, which might negatively affect the electrochemical active area of the electrode itself.

The EASA value obtained for the best performing electrode, i.e., USC PEDOT 100, shows an α value of (2.6 ± 0.2).

### 3.3. Electrodes Operation in MFCs

#### 3.3.1. MFC Output Potential Monitoring

**Figure 8:**
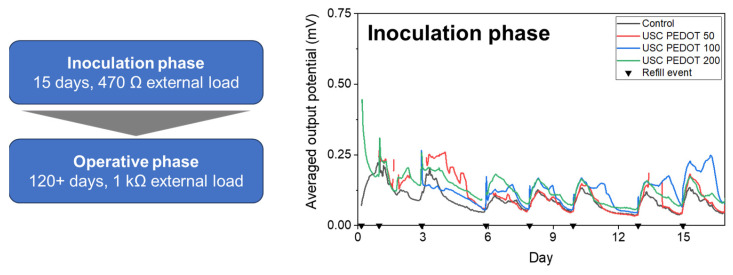
Average performance recorded on MFCs during the initial inoculation period [33]. Each black triangle represents one refill event with inoculum medium.

Figure 8 reports the average potential output, generated by each MFCs’ triplet during the initial inoculation phase. All results allowed us to confirm the successful biofilm growth on all anode electrodes’ surface, with MFCs providing a stable voltage trend already after one week from the beginning of the experiment. In particular, it was possible to highlight how the presence of the PEDOT:PSS nanostructured layer on the anodes’ surface positively affected the biofilm formation. 

**Figure 9:**
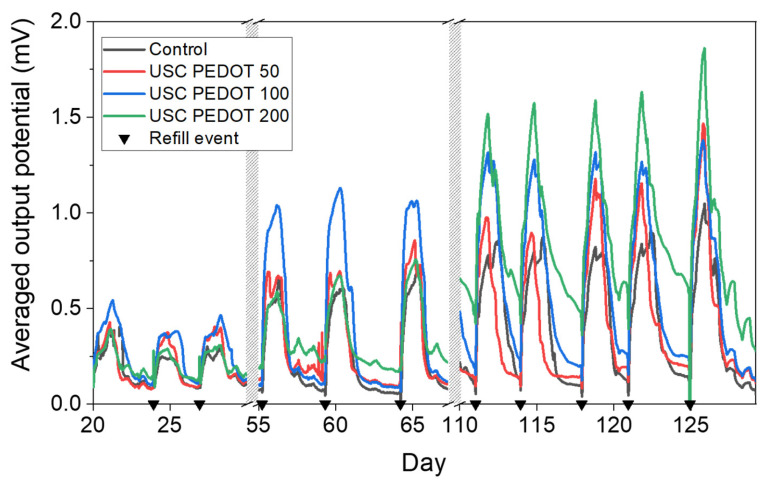
Average performance computed over each triplet of MFC devices employed during the operative phase. Each black triangle represents one refill event with 1 g/L sodium acetate electrolyte solution.

The overall performance of each MFC is represented in Figure 9 in terms of voltage trends versus time. It is possible to observe an improvement in the performance when PEDOT:PSS layers were deposited on a carbon backbone. In particular, MFCs featuring the PEDOT:PSS layer reached potential maxima of (1.1 ± 0.3) mV (USC PEDOT 50), (1.31 ± 0.06) mV (USC PEDOT 100), and (1.6 ± 0.2) mV (USC PEDOT 200). Such values are almost twice as much as the voltage reached when bare carbon paper was involved as an anode electrode, which is equal to (0.9 ± 0.1) mV. Moreover, by analysing the voltage output achieved in general by the USC PEDOT anode electrodes in MFCs, it was possible to confirm an improvement of 45% in overall devices’ performance with respect to what was achieved with bare carbon paper anodes. 

In detail, comparing the performance of the devices when USC PEDOT 50, USC PEDOT 100, and USC PEDOT 200 are employed as anode electrodes, it can be noticed that the presence of the highest quantity of PEDOT, equal to 200 μg/cm^2^, does not lead to a significant increase in overall devices’ performance. 

Moreover, when analysing the peaks reached when USC PEDOT 50 and USC PEDOT 100 were employed as anodes, it was possible to observe that both higher potentials and a longer electrical activity duration were achieved.

To accurately quantify the performance improvement of SCMFCs featuring USC-deposited PEDOT:PSS, the E_rec_ parameter was analysed. E_rec_ values are reported in Table 2. It is possible to observe how, with respect to control cells, a higher energy recovery was systematically obtained from SCMFCs with the new USC-processed electrodes. Specifically, USC PEDOT 100 and USC PEDOT 200 provided an energy recovery close to (11.7 ± 0.9) J·m^−3^ and (13 ± 2) J·m^−3^, respectively. These values are three-times higher than the one reached by control cells, equal to (4.3 ± 0.5) J·m^−3^. Concerning the energy recovery parameter, it is possible to appreciate a slight improvement, when the PEDOT:PSS amount increased from USC PEDOT 100 to USC PEDOT 200. 

This consideration, combined with the analysis of output potential trends over time, allowed us to state that the presence of the PEDOT:PSS nanostructured layer on the carbon backbone effectively improved the performance of bio-electrochemical devices. At the same time, it was possible to conclude that an increase in the PEDOT amount might be excessive, as the deposition of 200 μg/cm^2^ did not lead to significant improvements, in terms of voltage output and energy recovery, with respect to 100 μg/cm^2^. 

#### 3.3.2. Electrochemical Characterizations on MFCs

**Figure 10:**
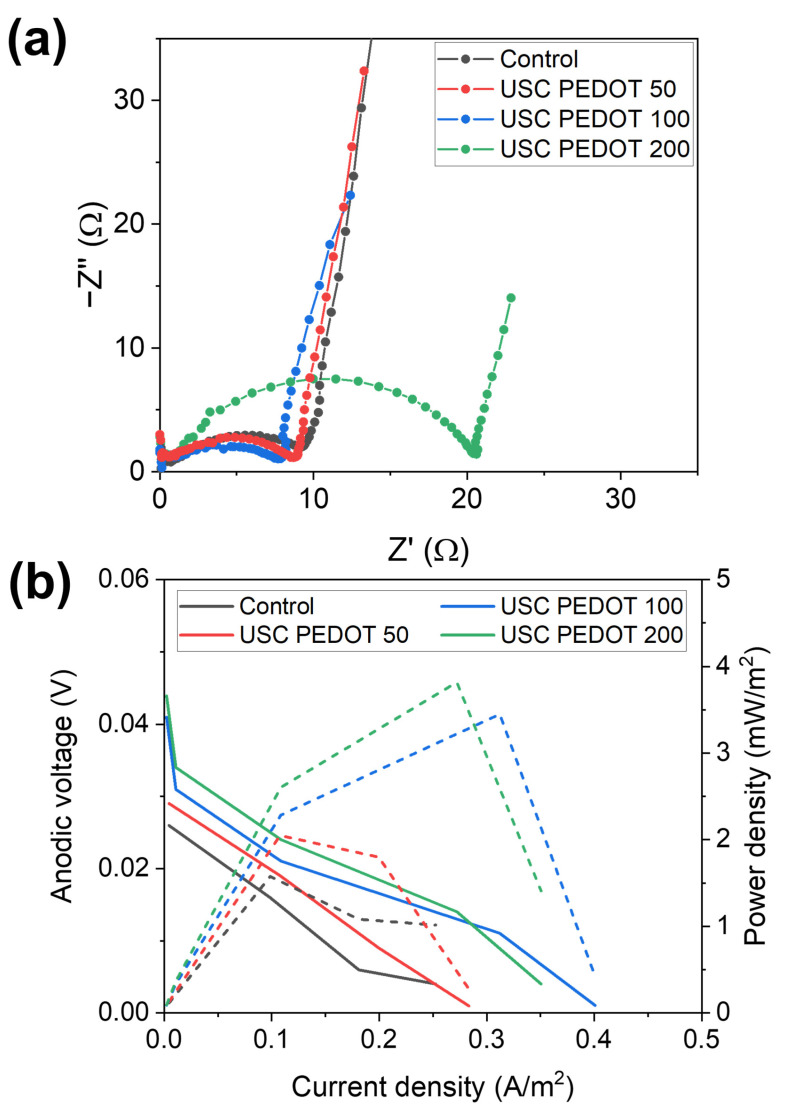
Comparison of (**a**) EIS spectra and (**b**) LSV polarization (solid lines, left axis) and power density (dashed lines, right axis) curves of one representative cell from each triplet of SCMFCs.

The abovementioned statement was confirmed by all results achieved by performing EIS and LSV characterizations. Figure 10a reports Nyquist plots obtained for all SCMFCs [39,40,41,42,43,44], which featured USC PEDOT 50, USC PEDOT 100, and USC PEDOT 200 as anode electrodes, to be compared with the control represented by bare carbon paper. From spectra acquired with a three-electrodes configuration, an important parameter is represented by the charge transfer resistance (R_ct_) element. This is associated to two interfaces, namely the electrode–biofilm and biofilm–electrolyte interfaces, which overlap one another in that frequency range. Values obtained from fitting EIS spectra, averaged for each triplet, are reported in Table 2 as the R_ct_ anode.

It is possible to observe how all R_ct_ values are compatible among all MFCs triplets, with the sole exception of the USC PEDOT 200 triplet, which presented a slightly increased R_ct_ value, equal to (19 ± 2) Ω. This higher value might indicate a thicker biofilm formation on the anode electrode, and leads us to confirm that 200 μg/cm^2^ of deposited PEDOT did not play an active role in enhancing the overall MFCs’ performance. On the contrary, USC PEDOT 100 provided a noticeable improvement in power output and energy recovery values with respect to bare carbon paper, without significantly affecting the R_ct_ value.

Figure 10b displays LSV characterization, with solid lines representing polarization curves (left axis) and dashed lines (right axis) the power density curves. From LSV characterizations, it was possible to confirm the trend observed in previous characterizations. Indeed, the performance of carbon paper electrodes was significantly increased by USC PEDOT 100 and USC PEDOT 200 electrodes. Nonetheless, no significant improvement can be observed when increasing the PEDOT:PSS amount above 100 μg/cm^2^.

Considering open circuit potentials, the measured values were 26 mV (Control), 29 mV (USC PEDOT 50), 41 mV (USC PEDOT 100), and 44 mV (USC PEDOT 200).

In terms of the short circuit current, USC PEDOT 100 performed the best at 401 mA/m^2^, while USC PEDOT 200 provided 350 mA/m^2^. Instead, the current for USC PEDOT 50 was 283 mA/m^2^, slightly higher than the control cell providing 252 mA/m^2^.

## 4. Conclusions

This work presents ultrasonic spray coating as a promising technique to obtain nanostructured layer depositions for optimizing carbon-based electrodes to be applied as anodes in bio-electrochemical devices. In particular, the possibility to apply USC to water-based inks containing the intrinsically conductive polymer PEDOT:PSS was confirmed. All the results achieved allowed us to conclude that the nanostructure of the PEDOT:PSS layer obtained on the electrodes’ surface optimally covered the complex structure of carbon-based electrodes. This permitted us to combine the continuous electrical conductivity of PEDOT:PSS with the porosity of standard carbon-based materials. EDX characterizations and Raman spectroscopy confirmed the spatial distribution uniformity of PEDOT:PSS on the electrodes’ carbon backbone. This feature was pivotal in optimizing anodic surfaces to host and sustain electroactive biofilms. Herein, we demonstrated the focal role of PEDOT:PSS in increasing the electrochemical active surface area of the electrode, providing a more than two-fold increase with respect to bare carbon paper. Moreover, experiments performed employing the newly fabricated electrodes as anodes in SCMFC devices demonstrated the performance improvements provided by USC-optimized anodes. The experiments provided clear evidence that the best performance was achieved when USC PEDOT 100 electrodes were used as anodes in SCMFCs, without the necessity to further increase the amount of deposited PEDOT. All the results obtained in this work confirmed that the beneficial effects observed from PEDOT:PSS deposited by USC were related to the concentration of the conductive polymer. This result is also in agreement with what is proposed in the literature describing other deposition methods. 

In conclusion, the application of USC as a methodology to improve the performances of anodes in microbial fuel cells has been validated. USC results in a versatile technology for the fabrication of nano-coatings, with optimal uniformity and conformity, which can be easily scaled up for large area depositions.

## Figures and Tables

**Table 1:** Electrochemical active surface area (EASA) as obtained from CV characterization and Matsuda’s equation.

Electrode	EASA (cm^2^)	α (a.u.)
Control (carbon paper)	6.6 ± 0.3	1
USC PEDOT 50	15.9 ± 0.3	2.40 ± 0.04
USC PEDOT 100	17 ± 2	2.6 ± 0.2
USC PEDOT 200	15 ± 2	2.3 ± 0.3

**Table 2:** Calculated energy recovery for each triplet of MFC devices and R_ct_ values obtained by fitting the electrochemical impedance spectra and averaging over each triplet of cells. Values of R_ct_ were measured via a three-electrodes configuration.

MFC Device	E_rec_ (J·m^−3^)	R_ct_ Anode (Ω)
Control (carbon paper)	4.3 ± 0.5	11 ± 2
USC PEDOT 50	6.2 ± 0.6	10 ± 3
USC PEDOT 100	11.7 ± 0.9	11 ± 2
USC PEDOT 200	13 ± 2	19 ± 2

## Data Availability

Data are contained within the article.
